# Diverse actions of sirtuin-1 on ovulatory genes and cell death pathways in human granulosa cells

**DOI:** 10.1186/s12958-022-00970-x

**Published:** 2022-07-15

**Authors:** Jackson Sapuleni, Magdalena Szymanska, Rina Meidan

**Affiliations:** 1 Department of Animal Sciences, The Robert H. Smith Faculty of Agriculture, Food and Environment, The Hebrew University of Jerusalem, 761001 Rehovot, Israel; 2 Institute of Animal Reproduction and Food Research of the Polish Academy of Sciences, Tuwima 10, 10-748 Olsztyn, Poland

**Keywords:** Ovulation, Angiogenesis, Apoptosis, Necroptosis

## Abstract

**Background:**

Human granulosa-lutein cells (hGLCs) amply express sirtuin-1 (SIRT1), a NAD + -dependent deacetylase that is associated with various cellular functions. SIRT1 was shown to elevate cAMP on its own and additively with human chorionic gonadotropin (hCG), it is therefore interesting to examine if SIRT1 affects other essential hGLC functions.

**Methods:**

Primary hGLCs, obtained from the follicular aspirates of women undergoing IVF and SV40-transfected, immortalized hGLCs (SVOG cells), were used. Primary cells were treated with SIRT1 specific activator SRT2104, as well as hCG or their combination. Additionally, siRNA-targeting SIRT1 construct was used to silence endogenous SIRT1 in SVOG cells. *PTGS2*, *EREG*, *VEGFA* and *FGF2* expression was determined using quantitative polymerase chain reaction (qPCR). Apoptotic and necroptotic proteins were determined by specific antibodies in western blotting. Cell viability/apoptosis was determined by the XTT and flow cytometry analyses. Data were analyzed using student *t*-test or Mann–Whitney U test or one-way ANOVA followed by Tukey HSD *post hoc* test.

**Results:**

In primary and immortalized hGLCs, SRT2104 significantly upregulated key ovulatory and angiogenic genes: *PTGS2*, *EREG*, *FGF2* and *VEGFA*, these effects tended to be further augmented in the presence of hCG. Additionally, SRT2104 dose and time-dependently decreased viable cell numbers. Flow cytometry of Annexin V stained cells confirmed that SIRT1 reduced live cell numbers and increased late apoptotic and necrotic cells. Moreover, we found that SIRT1 markedly reduced anti-apoptotic BCL-XL and MCL1 protein levels and increased cleaved forms of pro-apoptotic proteins caspase-3 and PARP. SIRT1 also significantly induced necroptotic proteins RIPK1 and MLKL. RIPK1 inhibitor, necrostatin-1 mitigated SIRT1 actions on RIPK1 and MLKL but also on cleaved caspase-3 and PARP and in accordance on live and apoptotic cells, implying a role for RIPK1 in SIRT1-induced cell death. SIRT1 silencing produced inverse effects on sorted cell populations, anti-apoptotic, pro-apoptotic and necroptotic proteins, corroborating SIRT1 activation.

**Conclusions:**

These findings reveal that in hGLCs, SIRT1 enhances the expression of ovulatory and angiogenic genes while eventually advancing cell death pathways. Interestingly, these seemingly contradictory events may have occurred in a cAMP-dependent manner.

**Supplementary Information:**

The online version contains supplementary material available at 10.1186/s12958-022-00970-x.

## Background

It is widely accepted that changes initiated by luteinizing hormone (LH) in the preovulatory follicle, mainly in the granulosa cell (GC) layer, coordinate ovulation [1–3]. Acting primarily via cAMP, LH elevates factors that are essential for a successful ovulation, these include prostaglandin endoperoxide synthase 2 (PTGS2); the rate limiting enzyme of PGE2 and other prostaglandins [4, 5] and epidermal growth factor-like molecules: amphiregulin and epiregulin (EREG) causing cumulus expansion and oocyte maturation [6–8]. Along with ovulatory genes, LH induces two key luteal angiogenic factors: fibroblast growth factor 2 (FGF2) and vascular endothelial growth factor A (VEGFA) [9, 10]; expressed at particularly high levels during follicular–luteal transition.

An additional factor highly expressed in a cAMP-dependent manner in luteinized human GCs (hGLCs) is sirtuin-1 (SIRT1) [11–13]. SIRT1 is a NAD + -dependent deacetylase associated with various biological functions [14–16], including regulation of proliferation and secretory activity of GCs [17–20]. It was further demonstrated that SIRT1 elevated cAMP on its own and additively with human chorionic gonadotropin (hCG) [12], however whether SIRT1 affect ovulatory and angiogenic genes is yet to be examined.

To enhance enzymatic activity of SIRT1, several activators were investigated; the first and most frequently used is resveratrol, a polyphenol particularly abundant in grape skins [21, 22]. However, besides SIRT1 activation, resveratrol has antioxidant and anti-inflammatory actions [23, 24]. In addition, resveratrol stimulates other members of the SIRT family and is therefore a non-specific activator of SIRT1 [25, 26]. Consequently, specific and more potent synthetic activators of SIRT1 such as SRT1720 and SRT2104 were developed [27, 28]. We have previously reported that SRT2104 indeed augments SIRT1 levels in hGLCs and modulates cells’ functions. Amongst its action we observed that SRT2104 caused hGLCs’ death [19], but the detailed mechanism involved in SIRT1 –induced cells’ death remains unresolved.

A delicate balance between survival and apoptotic factors determines GCs’ fate. The BCL2 family proteins comprise a network that regulates intrinsic survival/apoptotic responses [29] and contains anti- and pro-apoptotic proteins. There are BCL2 homologs that promote cell survival such as B-cell lymphoma 2 (BCL2), B-cell lymphoma-extra-large (BCL-XL), and myeloid cell leukemia 1 (MCL1). Programmed cell death is executed by caspases, including caspase 3 which further cleaves poly(ADP-Ribose) polymerase (PARP) ultimately resulting in DNA fragmentation and apoptosis [30–34]. Another form of programmed cell death termed necroptosis was described [35, 36]. In this cell death pathway, receptor interacting protein kinase (RIPK) 1 and 3 cooperate through their RIP homotypic interaction motif, leading to the recruitment and activation of mixed lineage kinase domain like pseudokinase (MLKL), which is the executor of necroptosis [37, 38]. Interestingly, it was shown that RIPK1 can also promote apoptotic pathways [39, 40]. However, it still needs to be clarified whether one or more of these pathways are activated by SIRT1 in hGLCs.

To further understand the physiological relevance of SIRT1 in human GCs we sought here to determine how SIRT1 affects essential GCs’ functions, angiogenic and ovulatory genes as well as cell viability. To this end, SIRT1 levels were manipulated with specific activator, SRT2104, or with SIRT1 small interfering RNA (siRNA) constructs. Experiments were carried out with primary and immortalized hGLCs (SVOG cells).

## Materials and methods

### Chemical reagents

Unless otherwise stated, all biochemical reagents were obtained from Sigma-Aldrich (St. Louis, MO, USA) and cell culture materials were from Biological industries (Kibbutz Beit Haemek, Israel).

### Culture of primary and immortalized hGLCs

Granulosa-lutein cells were obtained from the follicular aspirates of four women under 35 years of age who were subjected to the long suppression protocol [41] by IVF due to male factor infertility, as previously described [19, 42]. At least ten follicles from each patient were used to obtain follicular fluids that were further combined and used for each cell isolation. Four aspirations from four women were performed to obtain GLCs for four separate experiments. Briefly, the aspirates were centrifuged (3 min at 3000 × g) and erythrocytes were removed using an Ammonium Chloride Potassium (ACK) buffer (0.15 mol/L NH4Cl, 1.0 mmol/L KHCO3, and 0.1 nmol/L EDTA). Moreover, owing to ethical issues and the limited number of primary hGLCs obtained during the isolation procedure, non-tumorigenic immortalized hGLCs also termed SVOG cells were also used in this study. These cells were a generous gift from N Auersperg and P Leung (University of British Columbia, Canada) produced by transfecting primary hGLCs with the SV40 large T antigen [43]. SVOG cells have produced comparable results as those obtained in primary cells demonstrating that they are a reliable model for hGLCs [19, 44–47]. In the current study, cells from passage 14–19 were utilized.

hGLCs were seeded in 6-well plates (150,000 cells/well), while SVOG cells were seeded in T25 culture flasks (600,000 cells/flask) and only after reaching confluence, were seeded in 6-well plates. Cells were cultured in DMEM/F-12 containing 10% fetal calf serum (FCS), 2 mmol/L L-glutamine and 100 mg/mL penicillin/streptomycin in a humidified atmosphere of 95% air and 5% CO_2_ at 37°C**.** SVOG cells were treated with control medium (DMEM/F-12 containing 1% FCS) or SRT2104 (50 μmol/L) for 24 h. Primary hGLCs were incubated with control medium, hCG (10 IU), SRT2104 (50 μmol/L), or the combined treatment of hCG and SRT2104 for 24 h. Then, the total RNA was extracted from the cells.

### siRNA transfection

SVOG cells were cultured for 24 h, and then transfected using INTERFERin transfection reagent (Polyplus, Illkirch, France) in Opti-MEM I Reduced Serum Medium at 1% FCS according to the manufacturer’s protocol. The cells were transfected with 10 nmol/L small interfering RNA (siRNA; Genecust, Luxembourg) constructs targeting SIRT1, or with scrambled siRNA (siNC; negative control). The SIRT1 siRNA (siSIRT1) sequences were UAAUCCUGAAAUUCUUAGC [dT][dT] (sense) and GCUAAGAAUUUCAGGAUUA [dT][dT] (antisense). The siNC sequences were UUCUCCGAACGUGUCACGUTT [dT][dT] (sense) and ACGUGACACGUUCGGAGAATT [dT][dT] (antisense). After 6 and 24 h of transfection, the media were replaced to DMEM/F-12 with 1% FCS. Cells were collected 48 h post-transfection for protein extraction.

### Determination of viable cell numbers

Total cell numbers were estimated as previously described [19, 48], using the XTT assay Kit (Biological Industries), which measures the reduction of a tetrazolium component by the mitochondria of viable cells. Briefly, SVOG cells were seeded in 96 well plates (6000 cells/well) and cultured overnight in DMEM/F-12 containing 10% FCS. The next day, the cells were transferred to starvation medium (0.5% BSA in 0.1% FCS) and treated with either control or SRT2104 (10 -50 μmol/L) for 24-72 h. Each treatment was performed in quadruplicate. On the day of measurement, XTT was added to the culture media according to manufacturer’s instructions. Cells were then incubated at 37 °C for 3–5 h. Afterwards, the absorbance was read at 450 nm (reference absorbance, 630 nm).

### Analysis of cell death by flow cytometry

Cell viability/apoptosis was determined using the Annexin V-FITC and propidium iodide (PI) double-staining apoptosis using the MEBCYTO Apoptosis Kit (Medical and Biological, Woburn, MA) as previously described [49]. Briefly, cells were incubated with either control media, SRT2104 (50 µmol/L) for 24 or 48 h or when indicated pre-treated with 20 µmol/L necrostatin-1 (Nec-1) for 2 h, or cells were transfected with 10 nmol/L of siNC or siRNA targeting SIRT1 and collected 48 h post transfection. Afterwards, cells were trypsinized and washed once with PBS. Then, cells were centrifuged and suspended in 500 μL of binding buffer, after which 10 μL of Annexin V- FITC and 5 μl of PI staining solution were added and incubated at room temperature for 10 min. Next, the mixture was analyzed using a BD C6 Accuri (Franklin Lakes, NJ, USA) plus flow cytometer. Data analysis was done using the BD C6 Accuri plus software.

### Total RNA isolation and real-time PCR

Total RNA was extracted using the TRI Reagent (Molecular Research Center, Cincinnati, OH, USA) in accordance with the manufacturer’s instructions. Total RNA (1 μg) was reverse transcribed by using the qScript cDNA synthesis kit (Quantabio, Beverly, MA, USA). Quantitative polymerase chain reaction (qPCR) was performed using the LightCycler 96 system with LightCycler 480 SYBR Green I Master (Roche Diagnostics, Indianapolis, IN, USA), as described previously [19]. The sequences of primers used for qPCR are listed in Table 1. Primers were developed using Oligo Primer Analysis Software (Molecular Biology Insights, Inc., Colorado Springs, CO, USA), based on the available human sequences. All primers were designed to span an intron to prevent amplification of genomic DNA, and have single-product melting curves, as well as consistent amplification efficiencies between 1.8 and 2.2. Additionally, as designed the Tm (°C) for all reaction ranged between 55.8 to 56.2 ^o^ C as also previously described [19, 50]. The threshold cycle (Ct) values of each sample were generated, and the relative abundance of mRNA was calculated as 2^−ΔCt^ = 2^−(Ct target gene–Ct housekeeping gene)^ [51]. Expression data were normalized against housekeeping beta-actin (*ACTB*).

**Table 1:** Sequences of primers used for qPCR

Gene Name	Sequence (5’-3’)	Accession no.
*ACTB*	f: CGGGACCTGACGGACTACCTC	NM_001100 (46)
	r: GCCATCTCCTGCTCGAAGTCC	
*PTGS2*	f: CCCTTCCTCCTGTGCCTGATGA	NM_000963
	r: GTGAAGTGCTGGGCAAAGAATG	
*EREG*	f: AGGAGGATGGAGATGCTCTG	NM_001432
	r: TGGATTGTCTTCTGTCTGAAC	
*FGF2*	f: GCCAGGTAACGGTTAGCACAC	NM_002006
	r: GGCTTCTTCCTGCGCATCC	
*VEGFA*	f: ATCGAGACCCTGGTGGACA	NM_001025366 (47)
	r: CCTCGGCTTGTCACATCTGC	

*Abbreviations:* *f* forward, *r* reverse

### Protein extraction and western blot analyses

Total proteins were extracted from cultured cells as described previously [12, 46]. Briefly, cells were isolated by scrapping cells in sample buffer (1 mol/L Tris–HCl (pH 6.8), 20% (v/v) glycerol, 4% (w/v) SDS and 0.0002% (w/v) bromophenol blue and 1% (v/v) β-mercaptoethanol) containing phosphatase inhibitor cocktail 1 (Sigma Aldrich, St. Louis, MO, USA). The samples were sonicated for 10–15 s using a Microson Ultrasonic Cell Disruptor (Heat Systems Inc, NY, USA) to complete cell lysis and shear DNA and heated at 95 °C for 5 min. The proteins were separated on 7.5–14% SDS-PAGE, and subsequently transferred onto nitrocellulose membranes (Tamar Laboratory supplies ltd, Israel). Non-specific binding sites were blocked with 5% skim milk in TBST (20 mmol/L Tris, 150 mmol/L NaCl, and 0.1% Tween 20; pH 7.6) for 1 h at room temperature. The membranes were further incubated overnight at 4 °C with the primary antibodies listed in Table 2. The next day, membranes were washed three times in TBST (10 min each) and incubated with alkaline peroxidase-conjugated donkey anti-rabbit IgG (Table 2) in 1% skim milk in TBST for 1.5 h at room temperature. After washing with TBST three times for 5 min, immune complexes were visualized by chemiluminescence procedure using EZ ECL kit (Biological industries). Images were captured with Image Lab software (BioRad, Inc., Hercules, CA, USA). The intensity of bands was quantified using Gel-Pro 32 Software (Media Cybernetics, Silver Spring, MD), and normalized to the levels of total p44/42 MAPK, used as internal control for protein loading.

**Table 2:** Details of antibodies used for western blot

Antibody target	Dilution	Species specificity	Source	Supplier	Catalog no.
MCL1	1:1000	Human	Rabbit	Cell signaling Technology (Danvers MA, USA)	CS-5453
BCL-XL	1:1000	Human	Rabbit	Cell signaling Technology	CS-2762
PARP	1:1000	Human	Rabbit	Cell signaling Technology	CS-9532
Full-length Caspase 3	1:1000	Human	Rabbit	Cell signaling Technology	CS-14220
Cleaved Caspase 3	1:1000	Human	Rabbit	Cell signaling Technology	CS-9664
RIPK1	1:1000	Human	Rabbit	Cell signaling Technology	CS-14993
MLKL	1:1000	Human	Rabbit	Cell signaling Technology	CS-3493
Total p44/42 MAPK	1:5000	Human	Rabbit	Millipore (Burlington, MA, USA)	ABS44
HRP-linked IgG	1:10,000	Anti-rabbit	Donkey	Jackson immunoResearch (West Grove PA, USA)	711–035-152

### Statistical analyses

All statistical analyses were performed using GraphPad PRISM v.6.0 (GraphPad Software, Inc., San Diego, CA, USA). Data are presented as mean ± SEM. All experiments were repeated at least three times with each repeat constituting cells obtained from different passages. The student *t*-test and the Mann–Whitney U test were used to evaluate the statistical significance of differences between two groups. One-way ANOVA followed by Tukey HSD post-hoc test was used to analyze the significance of differences within and between multiple groups. In all analyses, a value of *p* ≤ 0.05 was considered significant, and unless otherwise stated asterisks (* *p*˂0.05, ** *p*˂ 0.01, *** *p*˂ 0.001) or hashtags (## *p*˂ 0.01, ### *p*˂ 0.001) indicate significant differences from their respective controls.

## Results

### SRT2104 augmented expression of ovulatory and angiogenic genes

**Fig. 1:**
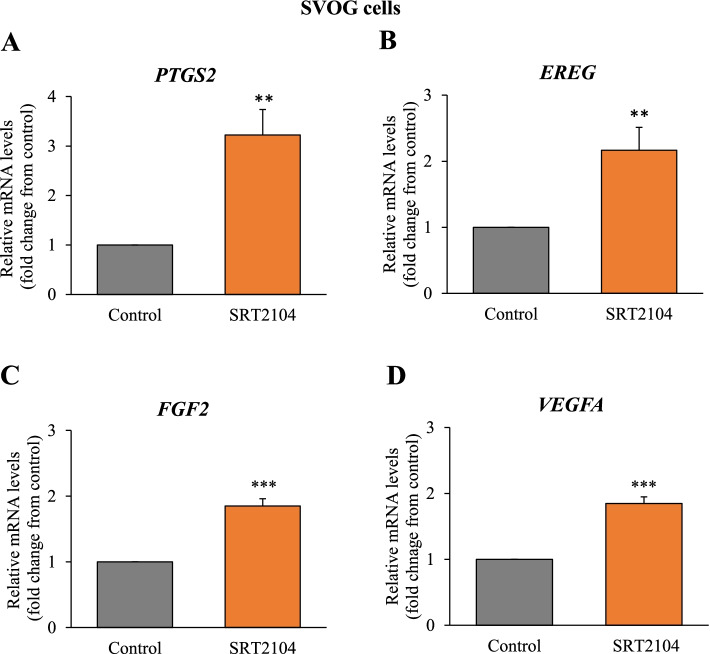
SRT2104 augments ovulatory and angiogenic genes in SVOG cells. Cells were incubated with either control media (designated as 1) or SRT2104 (50 μmol/L) for 24 h. Cells were then harvested, and mRNA expression was determined using qPCR. The results are presented as the means ± SEM of 3 independent experiments. Asterisks indicate significant (**p < 0.01 and ***p < 0.001) statistical differences from the control

**Fig. 2:**
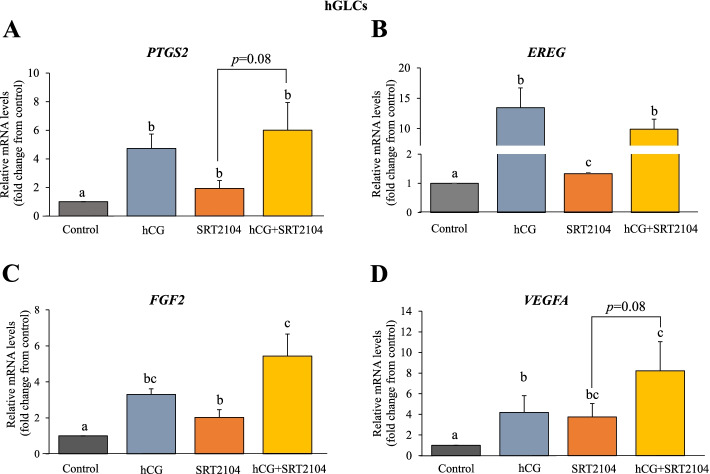
SRT2104 augments ovulatory and angiogenic genes in primary hGLCs. Cells were incubated with control medium alone, hCG (10 IU), SRT2104 (50 μmol/L), or the combined treatment of hCG and SRT2104 for 24 h. Cells were then harvested, and mRNA expression was determined using qPCR. The results are presented as the means ± SEM of 4 independent experiments. The different letters indicate significant statistical differences

Because SIRT1 was shown to elevate cAMP in hGLCs [12], it was of interest to investigate whether SIRT1 activator—SRT2104 affects key ovulatory and angiogenic genes. Indeed, we observed that SRT2104 significantly augmented expression levels of *PTGS2* (by 3.2-fold, *p* < 0.01, Fig. 1A), *EREG* (2.2-fold, *p* < 0.01, Fig. 1B), *FGF2* (1.9-fold, *p* < 0.001, Fig. 1C) and *VEGFA* (1.9-fold, *p* < 0.001, Fig. 1D) in SVOG cells. To examine the effect of SIRT1 in the presence of the physiological activator of cAMP in these cells –hCG, the next experiment utilized primary hGLCs (Fig. 2). A similar response was observed in the primary hGLCs; SRT2104 significantly stimulated these genes by 1.5—4 folds (Fig. 2). Interestingly, in these cells *VEGFA* was the gene most strongly affected by SIRT1 (Fig. 2D). As expected, hCG robustly stimulated *PTGS2*, *EREG*, *FGF2* and *VEGFA* (Fig. 2). The stimulatory effect of SRT2104 on *FGF2* was further significantly augmented in the presence of hCG; there was a similar tendency in the case of *PTGS2* and *VEGFA*. The combined treatment of hCG and SRT2104 also strongly increased *EREG* (7.5-fold) when compared to SRT2104 alone (1.3-fold; Fig. 2B), but there was no additive effect as compared to hCG alone, perhaps due to the robust EREG response to hCG alone in these cells.

### Effect of SIRT1 activation on viable and apoptotic cell populations

**Fig. 3:**
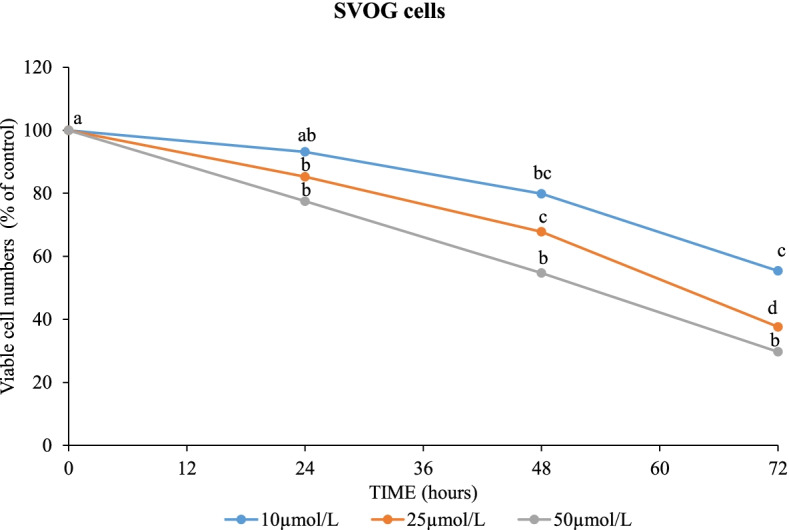
SRT2104 decreased viable cell numbers in a dose and time—dependent manner. SVOG cells were incubated with either control media or with varying concentrations (10, 25 or 50 μmol/L) of SRT2104 for 24, 48 and 72 h. Cell viability was determined using XTT assay. The results are presented as the means ± SEM of 3 independent experiments. The different letters indicate significant statistical differences at *p* < 0.05 analyzed using ANOVA followed by Tukey HSD post-hoc multiple comparison test

To further understand the physiological relevance of SIRT1 in human GCs, we evaluated its effect on cells’ viability. Different concentrations (10, 25 and 50 μmol/L) of SRT2104 were examined for various time points as indicated in Fig. 3. This data shows that SRT2104 significantly decreased viable cell numbers in a dose- and time-dependent manner with a maximal inhibitory effect of about 70% reduction after 72 h at the maximal dose examined (50 μmol/L) as compared to control cells. The number of control cells incubated in basal media remained stable over 72 h of culture (data not shown).

**Fig. 4:**
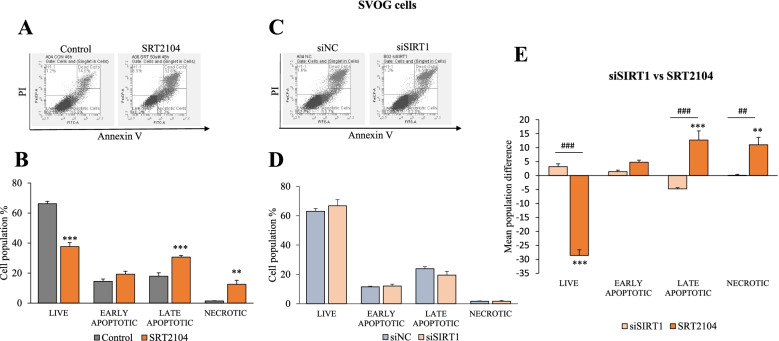
SRT2104 reduces live cells and increases late apoptotic and necrotic cells. **A** and **B** SVOG cells were treated with either control media or SRT2104 (50 μmol/L) or **C** and **D** cells were transfected with 10 nmol/L of either scrambled siRNA (siNC) or SIRT1 siRNA (siSIRT1). After 48 h post- treatment or transfection, cells were analyzed using FACS after propidium iodide (PI) and Annexin V staining (**A** and **C**). Results are presented as the means ± SEM from 4 and 3 independent experiments for SRT2104-treated and siSIRT1 transfected cells, respectively. **E** Comparison of mean cell populations between SIRT1 silencing and activation. The mean difference was calculated by subtracting the mean cell population of SRT2104- or siSIRT1- treated cells from their respective controls. Asterisks (**p < 0.01, ***p < 0.001) indicate significant differences between cells treated with SRT2104 or siSIRT1 compared to their respective controls. Hashtags (## *p* < 0.01, ### *p* < 0.001) indicate significant mean differences between cells treated with either SRT2104 vs siSIRT1

Next, we sought to support XTT results with flow cytometry which enables to detect apoptotic and necrotic cell populations (Fig. 4). Incubation of cells with 50 μmol/L of SRT2104 for 48 h resulted in significantly reduced live cell numbers and increased late apoptotic and necrotic cell populations compared to control cells (*p* < 0.01, Fig. 4B) confirming and extending XTT results.

To further substantiate the role of SIRT1, its endogenous levels were silenced using SIRT1 siRNA constructs. Transfection of SVOG cells with SIRT1 siRNA efficiently silenced SIRT1 mRNA (~ 75% decrease) and protein (~ 80% decrease) levels (data not shown, [12]). When compared to scrambled siRNA (siNC), SIRT1 silencing had no significant effects as shown in Fig. 4D. However, when SIRT1 silencing was compared to SRT2104 treatment (Fig. 4E), the opposing effects of activating and silencing SIRT1 on live, apoptotic and necrotic cell populations became evident. Knockdown of SIRT1 resulted in significantly greater number of live cells (*p* < 0.001) and fewer late apoptotic (*p* < 0.001) and necrotic cells (*p* < 0.01) as compared to SRT2104-treated cells.

### Effects of SIRT1 on pro- and anti- apoptotic proteins

**Fig. 5:**
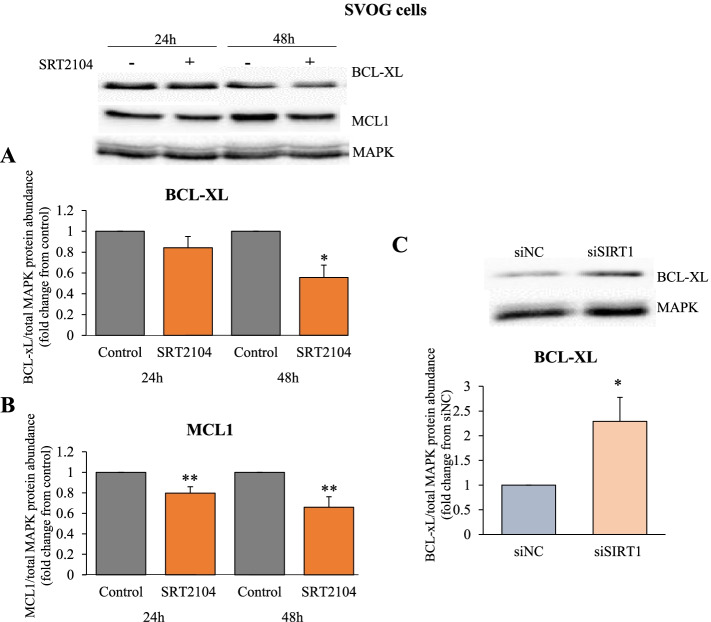
SIRT1 reduces anti-apoptotic proteins, BCL-XL and MCL1. **A** and **B** SVOG cells were treated with either control media or SRT2104 (50 μmol/L) for 24 h and 48 h. **C** Cells were transfected with 10 nmol/L of either scrambled siRNA (siNC) or SIRT1 siRNA (siSIRT1) and collected 48 h post transfection. BCL-XL and MCL1 protein levels were determined in cell extracts by western blotting. Cropped images representative of western blot are shown in the upper panels. Densitometric quantifications are relative to cells cultured in respective control media; protein levels were normalized to the abundance of total MAPK (p44/42; loading control). The results are presented as the means ± SEM from 3 independent experiments. Asterisks indicate significant differences from their respective controls (*p < 0.05, **p < 0.01)

To further elucidate the specific pathways responsible for SIRT1- induced cell death, we assessed anti-apoptotic members of BCL2 family: BCL-XL and MCL1. Significant effects were evident at 48 h following SRT2104 treatment where the levels of BCL-XL protein were significantly reduced by 45% (*p* < 0.05, Fig. 5A). MCL1 protein was significantly reduced as early as 24 h (20% reduction) and was further reduced after 48 h (35% reduction) of SRT2104 treatment (*p* < 0.01, Fig. 5B). These results were corroborated with SIRT1 silencing, showing that SIRT1 ablation was accompanied by a significant 2.3 fold increase in BCL-XL protein level (*p* < 0.05, Fig. 5C).

**Fig. 6:**
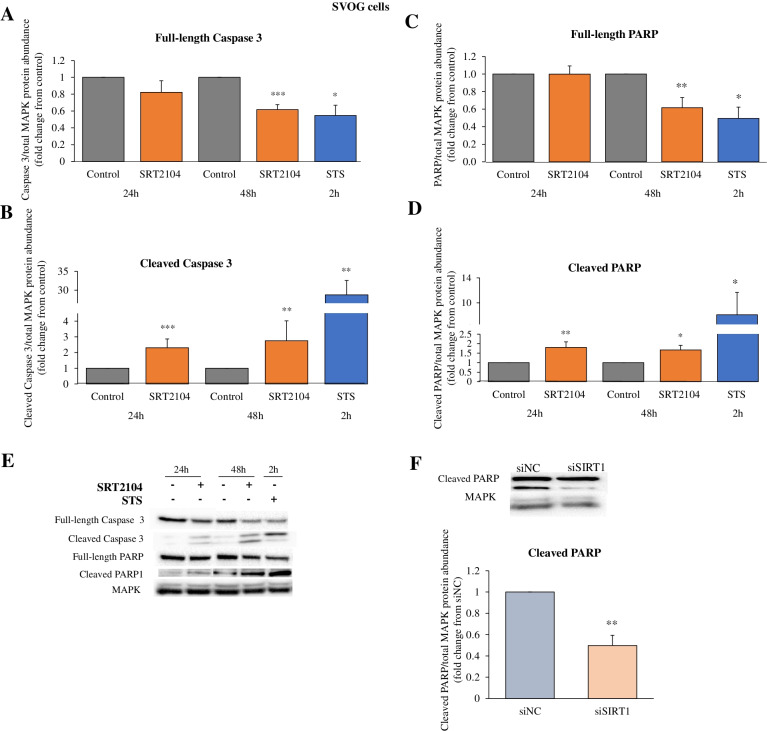
SIRT1 increases cleaved caspase 3 and PARP while decreasing full-length forms of these pro-apoptotic proteins. **A**-**D** Cells were treated with either control media or SRT2104 (50 μmol/L) for 24 h and 48 h or with Staurosporine (STS, 50 nmol/L) for 2 h (**A**-**D**). **F** Cells were transfected with 10 nmol/L of either scrambled siRNA (siNC) or SIRT1 siRNA (siSIRT1). At 6 and 24 h post-transfection, the media were changed, and protein was extracted at 48 h post-transfection. Full-length caspase 3 and cleaved caspase 3 protein levels (**A** and **B**, respectively), full-length PARP and cleaved PARP (**C** and **D**, respectively), were determined in cell extracts by specific antibodies in western blotting; cropped images representative of western blots are presented (**E**). Densitometric quantifications are relative to cells cultured in control media or transfected with siNC and normalized relative to the abundance of total MAPK (p44/42; loading control). The results are presented as the means ± SEM from 4 independent experiments. Asterisks indicate significant differences from their respective controls (*p < 0.05, ** *p* < 0.01, ***p < 0.001)

We then went on to investigate the effects of SIRT1 on pro-apoptotic proteins, full-length and cleaved caspase 3 as well as full-length and cleaved PARP. Full-length caspase 3 protein levels were significantly reduced (by 39%, *p* < 0.001) by SRT2104 only after 48 h treatment (Fig. 6A), while cleaved caspase 3 levels were markedly elevated from 24 h onwards; there was a 2.3-fold increase at 24 h and a further 2.8-fold increase at 48 h, as compared with the respective control levels (Fig. 6B). Similarly, full-length PARP protein expression was diminished by SIRT1 activation at 48 h treatment (*p* < 0.01, Fig. 6C) whereas cleaved and active PARP levels were significantly increased both at 24 h (1.8-fold increase, *p* < 0.01) and 48 h (1.7-fold increase, *p* < 0.05) as shown in Fig. 6D. In accord, SIRT1 silencing led to a significant reduction of 50% in cleaved PARP protein levels (*p* < 0.01, Fig. 6F). As a positive control we used staurosporine (STS) which is a broad-spectrum protein kinase inhibitor and effector of apoptosis in many cell types [52]. STS showed the expected reduction in the levels of full-length caspase 3 (*p* < 0.05, Fig. 6A) and full-length PARP (*p* < 0.05, Fig. 6C) with simultaneous robust elevation in cleaved caspase 3 (*p* < 0.01, Fig. 6B) and cleaved PARP (*p* < 0.05, Fig. 6D) proteins.

Moreover, we found that the ratio of cleaved to full-length caspase 3 levels were markedly elevated from 24 h onwards; there was a 4.6-fold increase at 24 h and a further 6.1-fold increase at 48 h, as compared with the respective control levels (Supplementary Fig. 1A). Similarly, the ratio of cleaved to full-length PARP levels were significantly increased both at 24 h (1.7-fold increase) and 48 h (2.3-fold increase) as shown in Supplementary Fig. 1B. SIRT1 silencing significantly reduced the ratio of cleaved to full-length PARP protein levels to 0.45 (*p* < 0.05, Supplementary Fig. 1C). These findings support the flow cytometry analyses presented in Fig. 4, confirming the role of SIRT1 as an activator of apoptotic pathway in hGLCs.

### Effects of SIRT1 on necroptosis

**Fig. 7:**
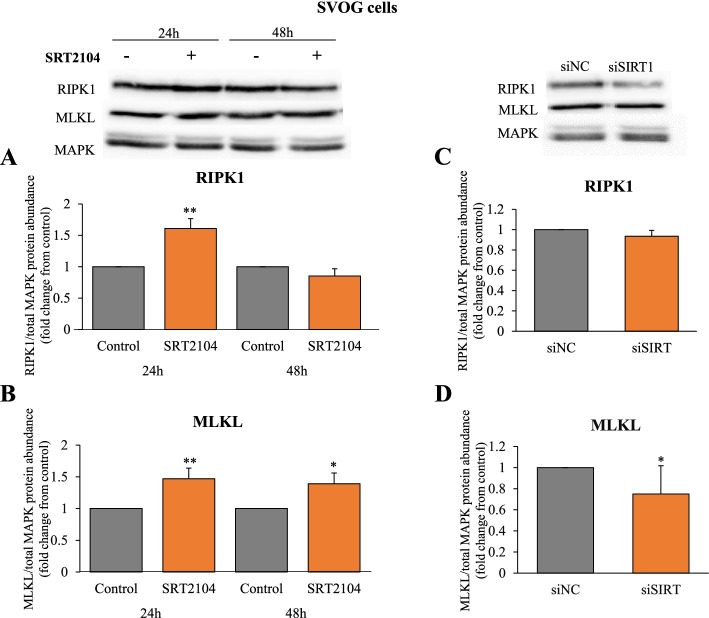
SIRT1 elevates necroptotic proteins RIPK1 (**A**,**C**) and MLKL (**B**,**D**). SVOG cells were treated with either control media or SRT2104 (50 μmol/L) for 24 h and 48 h (**A** and **B**) or cells were transfected with either scrambled siRNA (siNC) or SIRT1 siRNA (siSIRT1) and the protein was extracted at 48 h post-transfection (**C** and **D**). RIPK1 and MLKL protein levels were determined in cell extracts by specific antibodies in western blotting and normalized relative to the abundance of total MAPK (p44/42; loading control). Cropped images representative of western blot are shown in the upper panels. Results are presented as the means ± SEM from 4 independent experiments. Asterisks indicate significant differences from their respective controls (*p < 0.05, **p < 0.01)

Since an increase in necrotic cells after SRT2104 treatment was observed (Fig. 4A and B), we examined further the potential role of SIRT1 on necrosome components, RIPK1 and MLKL (Fig. 7). RIPK1 protein was significantly induced by SRT2104 at 24 h (Fig. 7A). MLKL protein abundance was also augmented by SIRT1 activation and this effect was evident from 24 to 48 h of incubation (1.5 and 1.4 **-**fold increase respectively, Fig. 7B). SIRT1 silencing, on the other hand, significantly reduced MLKL protein (*p* < 0.05, Fig. 7D), substantiating the role of SIRT1 in regulating this protein. However, SIRT1 knockdown had no effect on RIPK1 (Fig. 7C).

**Fig. 8:**
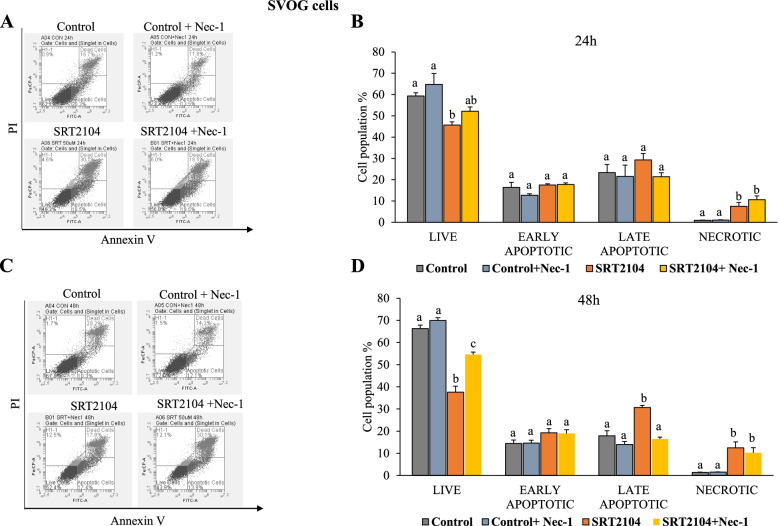
Nec-1 abolishes SRT2104 actions on live and late apoptotic cell populations. SVOG cells were pretreated with Nec-1 (20 μmol/L) for 2 h, followed by incubation for 24 (**B**) and 48 h (**D**) with medium only (control) or SRT2104 (50 μmol/L). Then, cells were analyzed using FACS with propidium iodide (PI) and Annexin V staining (representative plots are shown in A-24 h and C-48 h). Results are presented as the means ± SEM from 3 independent experiments. The different letters indicate significant statistical differences at *p* < 0.05 analyzed using ANOVA followed by Tukey HSD post hoc multiple comparison test

To further ascertain the potential role of necroptosis in SIRT1-induced hGLCs’ death, we assessed the outcome of a RIPK1 inhibitor, Nec-1 [53]. As shown in Fig. 8B, at 24 h Nec-1 did not significantly affect SRT2104 actions on either cell population. However, after 48 h of incubation, Nec-1 abolished SRT2104 actions on live and late apoptotic cell populations but had no effect on the necrotic cells as shown in Fig. 8D.

**Fig. 9:**
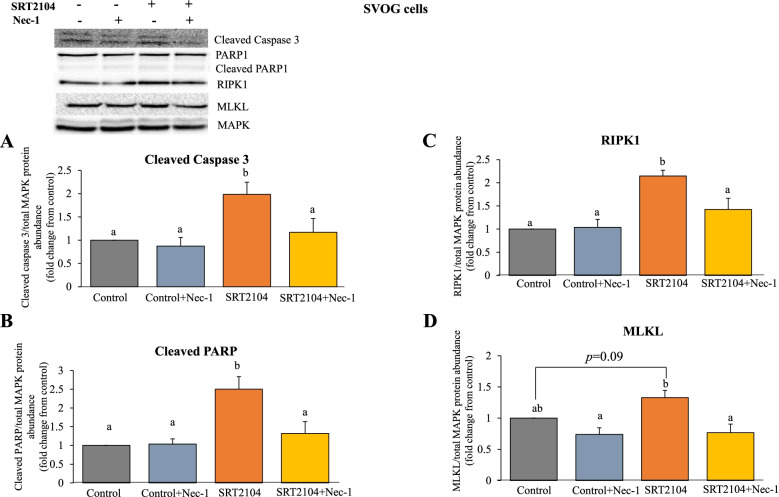
Nec-1 counteracts SIRT1 actions on pro-apoptotic and necroptotic proteins. SVOG cells were treated with either control media or SRT2104 (50 μmol/L) for 24 h or pretreated with Nec-1 (20 μmol/L) for 2 h. Protein levels (**A**- cleaved caspase 3, **B**- cleaved PARP, **C**-RIPK1 and **D**-MLKL) were determined in cell extracts by western blotting and normalized relative to the abundance of total MAPK (p44/42; loading control). Cropped image of representative western blots is shown in the upper panel. Results are presented as the means ± SEM from 3 independent experiments. The different letters indicate significant statistical differences at *p* < 0.05 analyzed using ANOVA followed by Tukey HSD post hoc multiple comparison test

We also examined the effect of Nec-1 on pro-apoptotic and necroptotic proteins in SRT2104-treated cells (Fig. 9). In agreement with the results presented in Fig. 8, Nec-1 significantly mitigated SRT2104-induced cleaved caspase 3 (41% reduction, Fig. 9A) and cleaved PARP (47% reduction, Fig. 9B), confirming that Nec-1 counteracts apoptotic actions of SIRT1. However, despite the observation that Nec-1 did not significantly affect SIRT1 induced necrotic cell population (in Fig. 8B and D), it counteracted SRT2104 induced RIPK1 and MLKL protein levels (33.6 and 42% reduction, respectively; Fig. 9C and D).

## Discussion

**Fig. 10:**
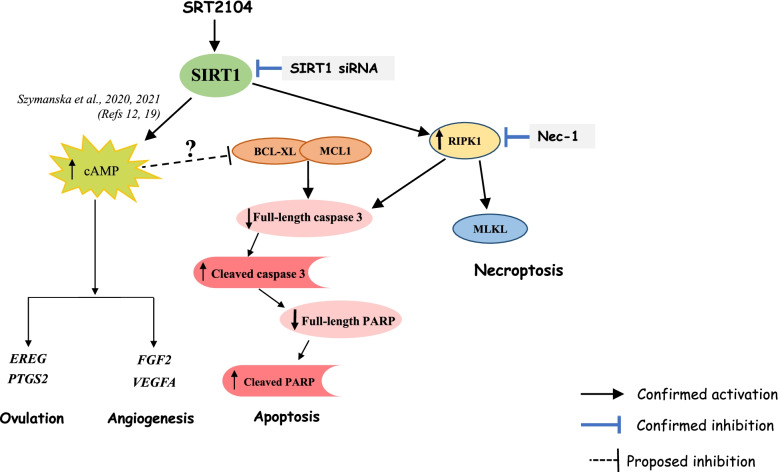
Illustrative summary depicting diverse actions of SIRT1 in human granulosa cells. SIRT1, activated by SRT2104 was previously shown to elevate cAMP levels [12]. SIRT1 induced genes pivotal for ovulation (*EREG* and *PTGS2*) and angiogenesis (*FGF2* and *VEGFA*), most likely in a cAMP-dependent manner. SIRT1 activation and subsequent elevation of cAMP may also promote reduction of anti-apoptotic proteins BCL-XL and MCL1. This triggers the cleavage and activation of caspase 3. Cleaved caspase 3 then cleaves PARP leading consequently to apoptosis. One may therefore suggest, SIRT1-induced cAMP is responsible for the coexistence of luteinization and apoptosis in luteinizing GCs. SIRT1 also activates RIPK1 and MLKL proteins thereby advancing necroptosis. Additionally, activation of RIPK1 can also mediate apoptosis by activating caspase 3. The role of RIPK1 in SIRT1 induced apoptosis and necroptosis is affirmed by the attenuation of apoptotic and necroptotic proteins by Nec-1. Pattern of FACS sorted cells further support the role of SIRT1 as inducer of apoptosis and necroptosis. Ablation of endogenous SIRT1 with siRNA produced opposing actions thereby corroborating the effects observed with SIRT1 activation

This study highlights the biological functions of SIRT1 in hGLCs, demonstrating that it upregulates the expression of key ovulatory genes *PTGS2* and *EREG,* and the main angiogenic genes *FGF2* and *VEGFA*. Several of these genes were further augmented in the presence of hCG suggesting cAMP-dependent action of SIRT1. This study also provides extensive data detailing the mechanisms of granulosa cell death initiated by SIRT1, showing that SIRT1 promotes both apoptosis and necroptosis (summarized in Fig. 10). Importantly, the effects of SIRT1 activation with SRT2104 were corroborated by SIRT1 silencing. These findings therefore indicate that SIRT1 exerts diverse and seemingly contradicting actions in hGLCs.

One of the novel findings in the present study is that SIRT1 upregulates the mRNA expression of *PTGS2*, *EREG*, *FGF2* and *VEGFA*, known to be elevated in GCs during the periovulatory period [4, 8, 54, 55], further studies are needed to ascertain that their protein levels are similarly elevated by SIRT1. These genes are elevated most probably as a result of SIRT1 ability to increase cAMP levels as previously observed in hGLCs [12] and in other cell types [56, 57]. However, unlike LH/hCG that stimulate cAMP by direct activation of adenylyl cyclase (AC), augmentation of cAMP by SIRT1 occurs via inhibition of phosphodiesterase enzymes which catalyze the breakdown of cAMP to inactive 5′AMP [58, 59]. This may be the reason why the magnitude of cAMP levels produced by SIRT1 activation [12] and consequently the increase of ovulatory and angiogenic genes in GCs (this study), are lower than those induced by LH/hCG [4, 8, 54, 55] and other upstream regulators of AC such as PGE2 [50]. Activation of two different mechanisms promoting intracellular cAMP levels can also explain the additive action of SIRT1 and hCG in hGLCs. This contention portrays SIRT1 as an auxiliary factor enhancing the expression of (ovulatory and angiogenic) genes whose role during the periovulatory period and corpus luteum (CL) formation is indispensable.

Studies have suggested that SIRT1 promotes apoptosis [60, 61], as also shown in the current study, where we observed that SIRT1 activation with SRT2104 dose- and time-dependently reduced viable cell numbers and increased late apoptotic and necrotic hGLC populations. Examining proteins considered to be hallmarks of apoptosis, the mechanism of SIRT1-induced cell death was revealed here. We found that SIRT1 reduced anti-apoptotic BCL2 proteins, BCL-XL and MCL1. Additionally, SIRT1 increased the levels of pro-apoptotic cleaved, active caspase 3 and PARP proteins. As well as the ratio of cleaved to full-length caspase 3 and PARP. Ablation of endogenous SIRT1 with siRNA produced opposing actions with increased live cell numbers and reduced late apoptotic and necrotic cell populations as compared to SIRT1 activation. SIRT1 silencing also led to increased BCL-XL protein and reduced the ratio of cleaved PARP to full-length PARP levels. Our results therefore suggest that SIRT1 promotes apoptosis by inhibiting anti-apoptotic BCL2 members and activating pro-apoptotic proteins.

Apart from apoptosis, a form of programmed necrosis termed necroptosis has also been unearthed. Necroptosis like necrosis, morphologically leads to rounding of the cell, cytoplasmic swelling and rupture of the plasma membrane thereby leading to spilling of the intracellular contents [35]. RIPK1, RIPK3 and MLKL are the essential regulators of necroptosis [37, 62–64]. Recently it has been suggested that necroptosis is one of the mechanisms of cell death in the ovary and studies have shown an increase in the necroptotic proteins RIPK1 and RIPK3 in cultured primary hGLCs [65, 66]. Also, the luteolytic hormone prostaglandin F2 alpha treatment increased protein levels of RIPK1, RIPK3 and MLKL in bovine CL [67, 68]. Here we showed that necrosome components RIPK1 and MLKL were elevated in hGLCs after SRT2104 treatment. These findings were further reinforced by the observation that silencing of SIRT1 led to reduced MLKL protein levels. We were however unable to detect phosphorylated forms of RIPK1 and MLKL at the time points employed, perhaps because phosphoproteins are unstable and may be difficult to detect. To the best of our knowledge, this is the first study highlighting the involvement of SIRT1 in necroptotic cell death in the ovary. Nec-1, a selective and potent allosteric inhibitor of RIPK1 [53, 69] further substantiated these findings. Nec-1 efficiently blocks RIPK1-RIP3-MLKL signal transduction thereby inhibiting necroptosis [53, 70]. Moreover, Nec-1 can also inhibit RIPK1- dependent apoptosis as it promoted cell survival and reduced the levels of apoptotic proteins in non-GC types [71, 72]. Accordingly, Nec-1 also increased the viability of luteal steroidogenic cells and hGLCs [66, 68]. Here we found that Nec-1 attenuated SIRT1 actions on live and late apoptotic GC populations and decreased SIRT1- induced apoptotic and necroptotic proteins, suggesting that SIRT1-induced necroptosis and apoptosis are RIPK1-dependent. Nec-1 did not however reduce the SIRT1 induced necrotic cell population in our study, while the time frame was sufficient for attenuating the proteins (which precede actual cell death) it was inadequate in preventing necroptosis.

Past evidence provided by many research groups including ours have convincingly demonstrated that SVOG cells responded similarly to primary hGLCs for a variety of signals including SIRT1[45, 73–76]. Therefore, the results obtained here on SIRT1 induced apoptosis and necroptosis in SVOG cells are relevant to primary hGLCs, however more research is necessary to demonstrate it unequivocally.

What is the mechanism underlying SIRT1 induced apoptosis? As discussed earlier we and others have demonstrated that SIRT1 activation enhances cAMP levels [12, 56, 57]. Perhaps surprisingly, quite a few studies had linked elevated intracellular levels of cAMP to apoptosis in various cell types [77–79] including ovarian follicular cells and GCs [80–83]. For instance, it was shown that treatment of GCs with forskolin, or 8-bromo-cAMP induced apoptosis as evidenced by DNA fragmentation and chromatin condensation. Death promoting effects of cAMP were also accompanied by an increase in the expression of apoptotic proteins [83]. In fact, Yacobi et al., reported that LH treatment induced in preovulatory follicles’ progesterone production and caspase activity [82, 84]. Our results may therefore suggest that SIRT1-induced cAMP is involved in promoting apoptosis of hGLCs.

## Conclusions

This study extends our knowledge of the roles and signaling pathways employed by SIRT1 in hGLCs during the periovulatory period. Together with our previous reports [12, 19], the results described here highlight the role of SIRT1 on critical GCs- borne factors, cAMP, endothelin 2, hypoxia-inducible factor 1 as well as key ovulatory and angiogenic genes, indicating that SIRT1 has wide-ranging effects on GCs functions.

## Supplementary Information

**Additional file 1. **

**Additional file 2: Supplemental Figure 1.** Ratios of cleaved to full length forms of caspase 3 and PARP. (A,B) SVOG cells were treated with either control media or SRT2104 (50 μmol/L) for 24h and 48h. (C) SVOG cells were transfected with 10 nmol/L of either scrambled siRNA (siNC) or SIRT1 siRNA (siSIRT1). The ratios between cleaved to full-length caspase 3 (A) and cleaved to full length PARP (B,C) were calculated from data presented in Figure 6. Asterisks indicate significant differences from their respective controls (*p < 0.05).

## Data Availability

The data presented in this study are available on personal request from the corresponding author.

## Abbreviations

AC: Adenylyl cyclase
BCL-XL: B-cell lymphoma-extra large
cAMP: Cyclic adenosine monophosphate
CL: Corpus luteum
EREG: Epiregulin
FGF2: Fibroblast growth factor 2
GCs: Granulosa cells
hCG: Human chorionic gonadotropin
hGLC: Human granulosa lutein cells
IVF: In vitro fertilization
LH: Luteinizing hormone
MCL1: Myeloid cell leukemia 1
MLKL: Mixed lineage kinase domain like pseudokinase
Nec-1: Necrostatin-1
PARP: Poly (ADP-ribose) polymerase
PI: Propidium iodide
PTGS2: Prostaglandin endoperoxide synthase 2
qPCR: Quantitative polymerase chain reaction
RIPK1: Receptor-interacting serine/threonine-protein kinase 1
siRNA: Small interfering RNA
SIRT1: Sirtuin-1
STS: Staurosporine
VEGFA: Vascular endothelial growth factor A
