# Supplementary material for: Diverse actions of sirtuin-1 on ovulatory genes and cell death pathways in human granulosa cells
Source: Reprod Biol Endocrinol. 2022 Jul 15;20:104. doi: 10.1186/s12958-022-00970-x (PMC9284863; doi:10.1186/s12958-022-00970-x)
Supplement: Supplementary file 1 — Additional file 1. [file 12958_2022_970_MOESM1_ESM.pdf]

Original western blots

In the additional files we have provided original unedited western blots images indicating the proteins we probed for. The highlighted sections, marked with red rectangles, in the original images indicate sections used to create final figures shown in this report. The calculations were based on each protein being normalized to its respective MAPK.

Figure 5

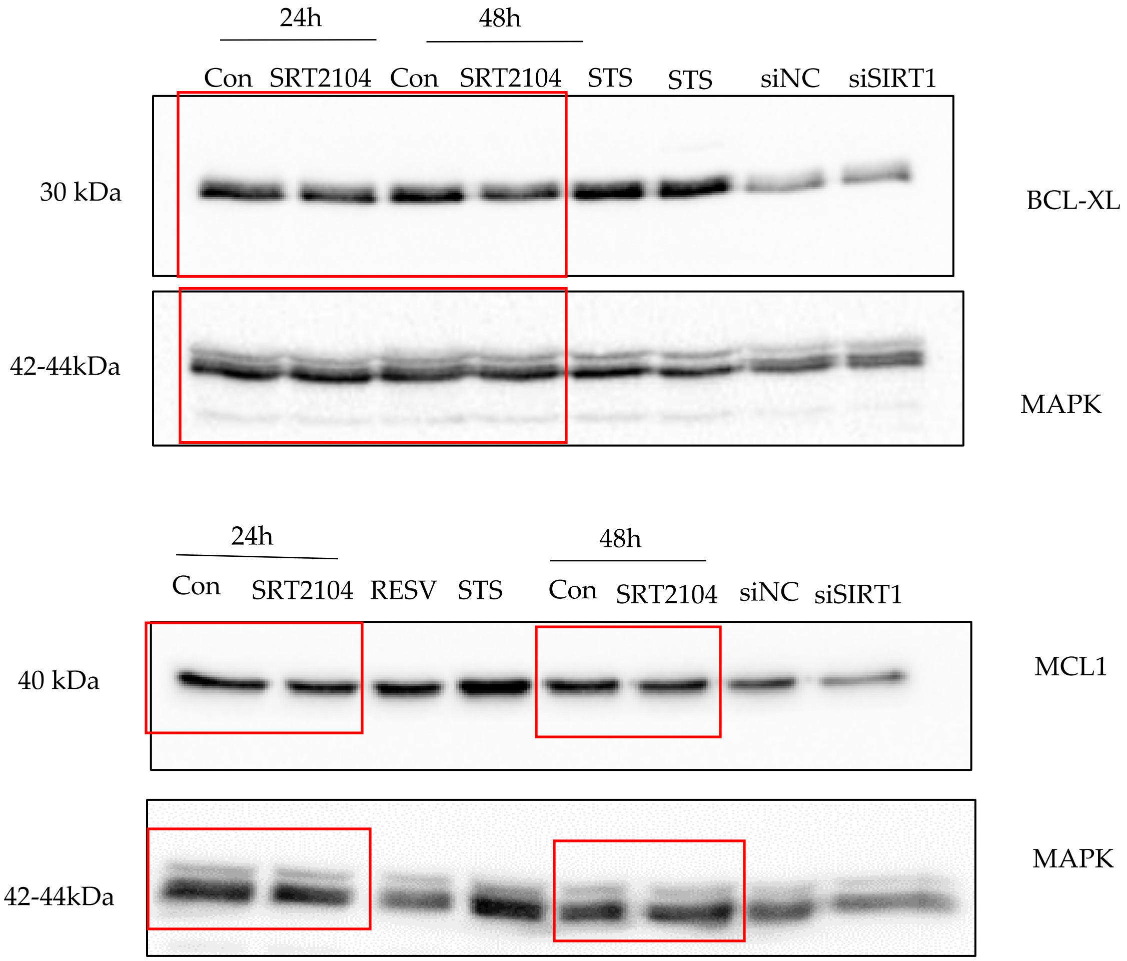

Figure 5C

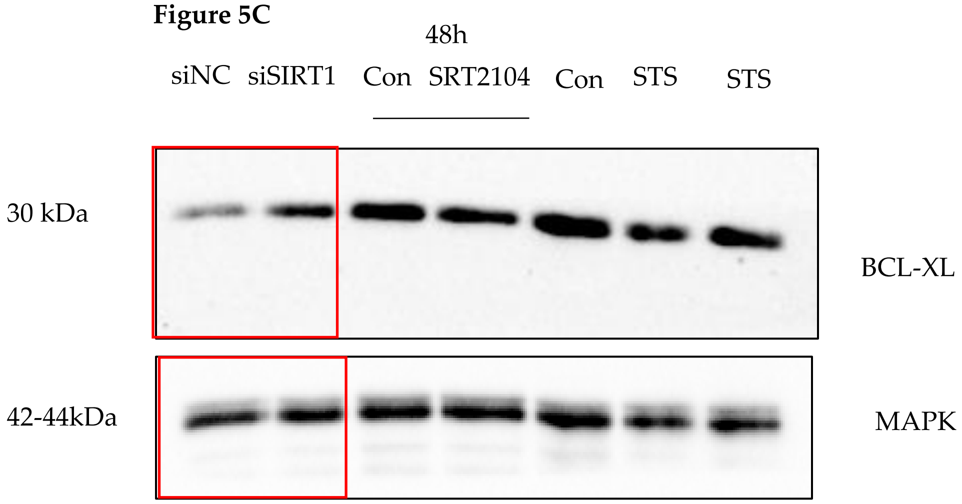

Figure 6E

In figure 6E, lane 3 was spliced out (since we do not show resveratrol here) and lanes 2 and 4 were joined in the final images shown in the report.

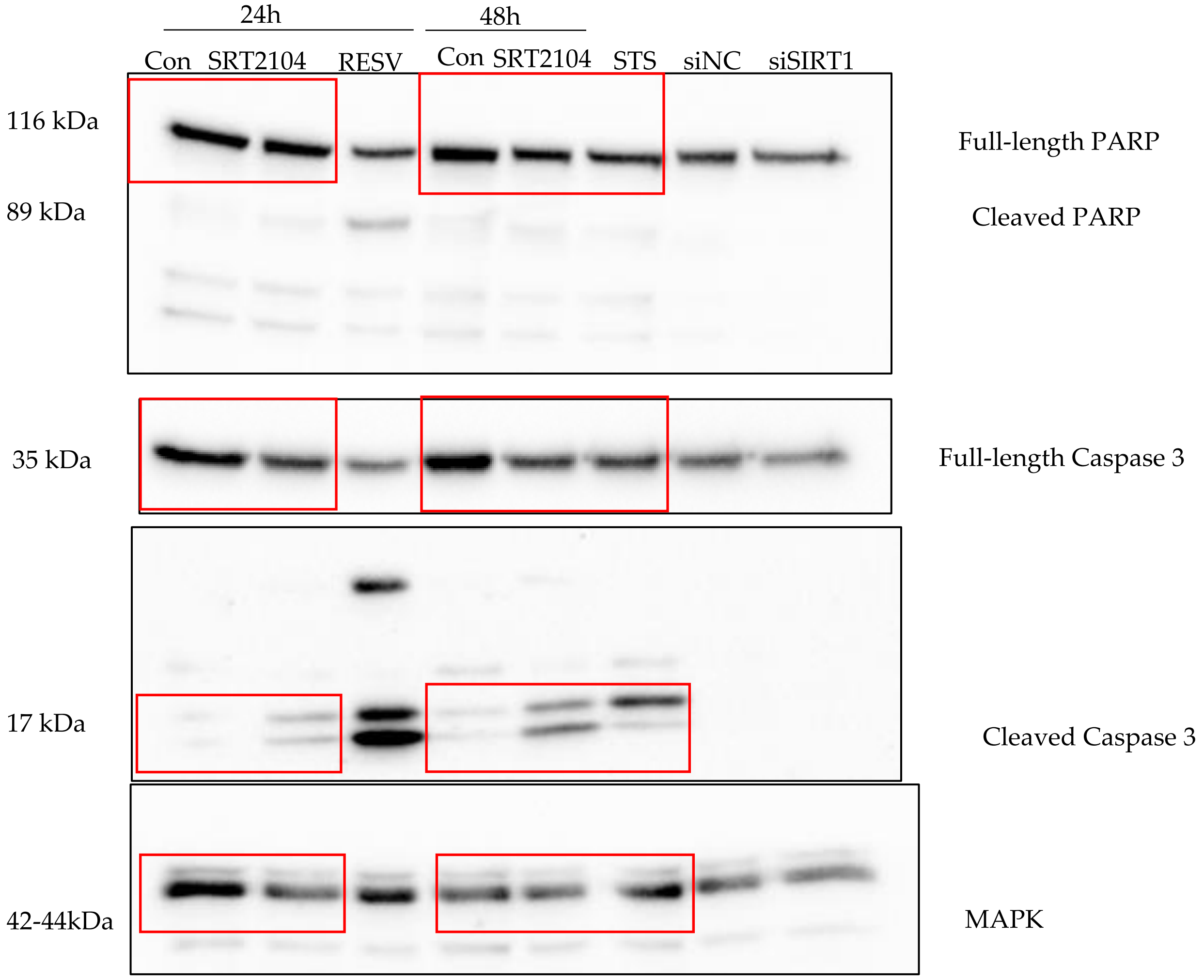

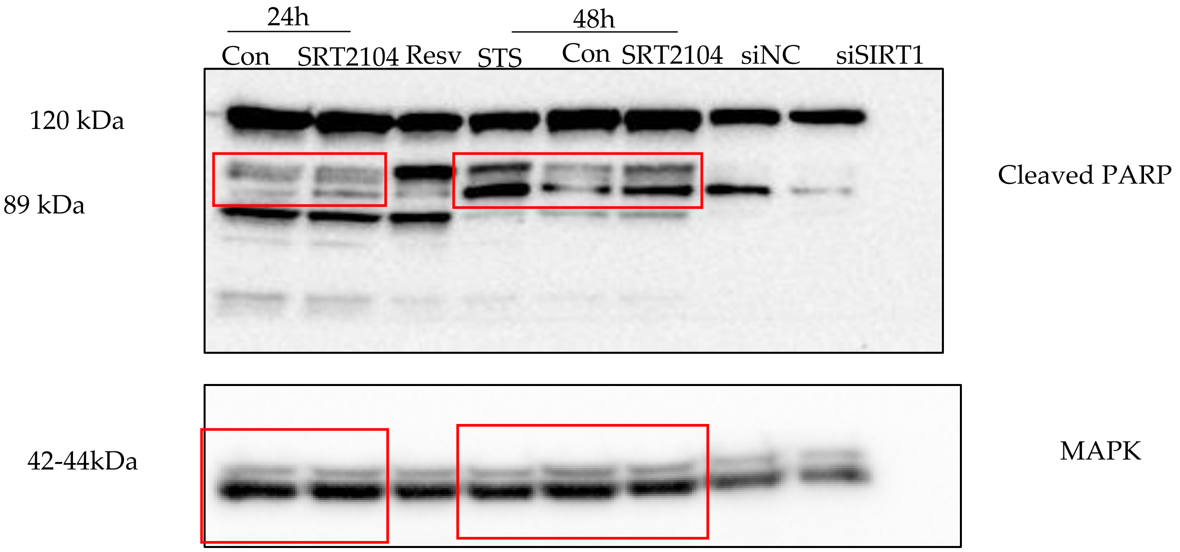
24h
48h
Con
SRT2104
Resv
STS
Con
SRT2104
siNC
siSIRT1
120 kDa
89 kDa
Cleaved PARP
42-44kDa
MAPK

Figure 6F

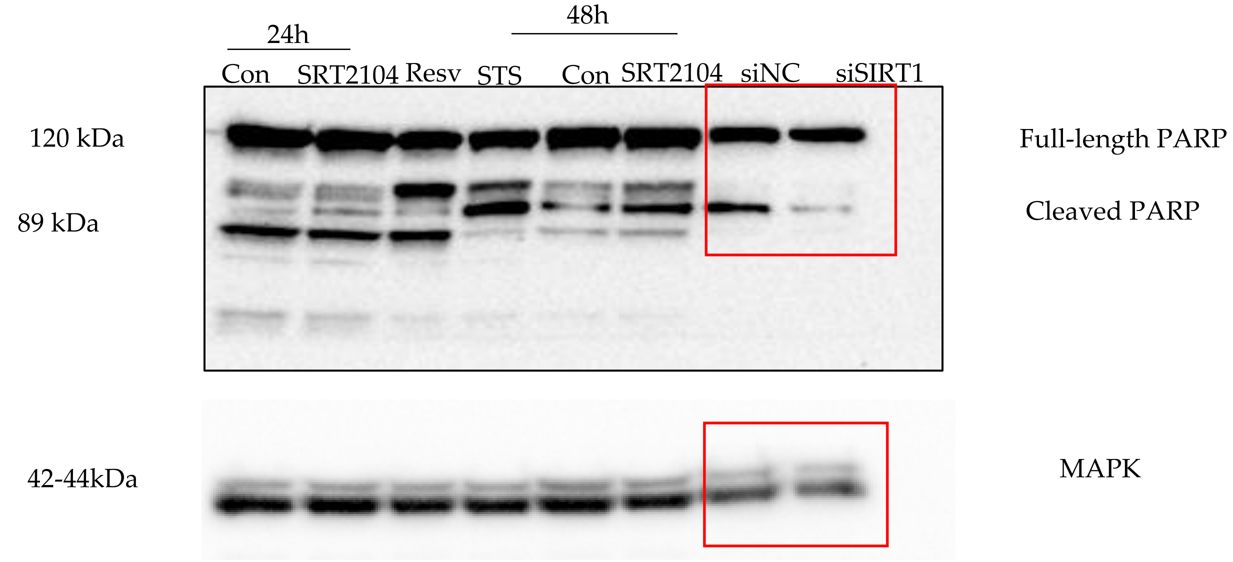
24h
48h
Con
SRT2104
Resv
STS
Con
SRT2104
siNC
siSIRT1
120 kDa
89 kDa
Full-length PARP
Cleaved PARP
42-44kDa
MAPK

Figure 7

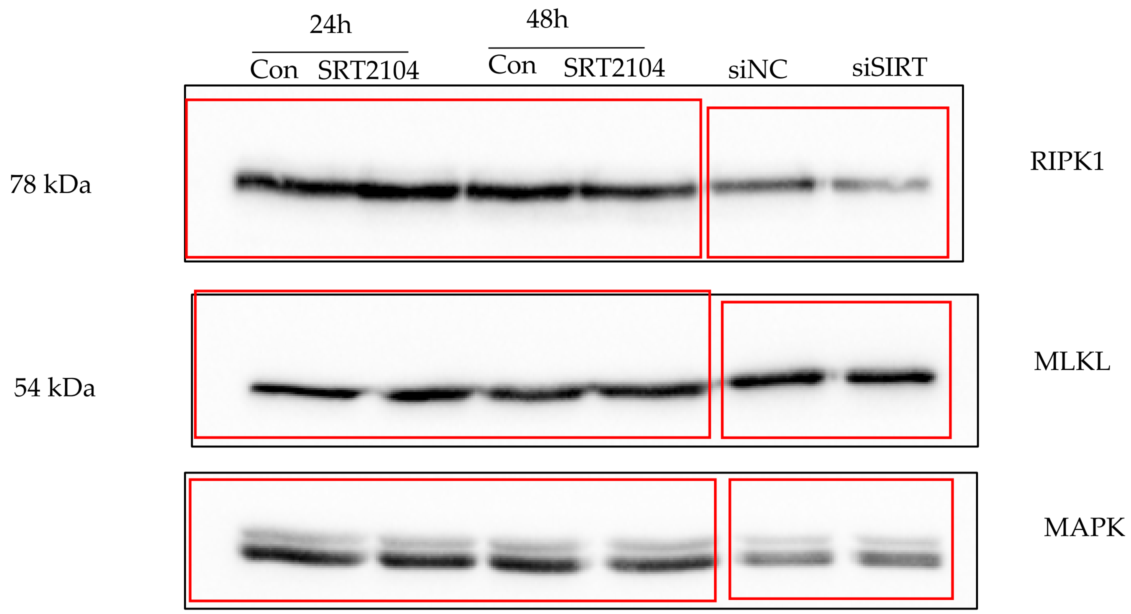
24h
48h
Con
SRT2104
Con
SRT2104
siNC
siSIRT
78 kDa
RIPK1
54 kDa
MLKL
MAPK

Figure 9

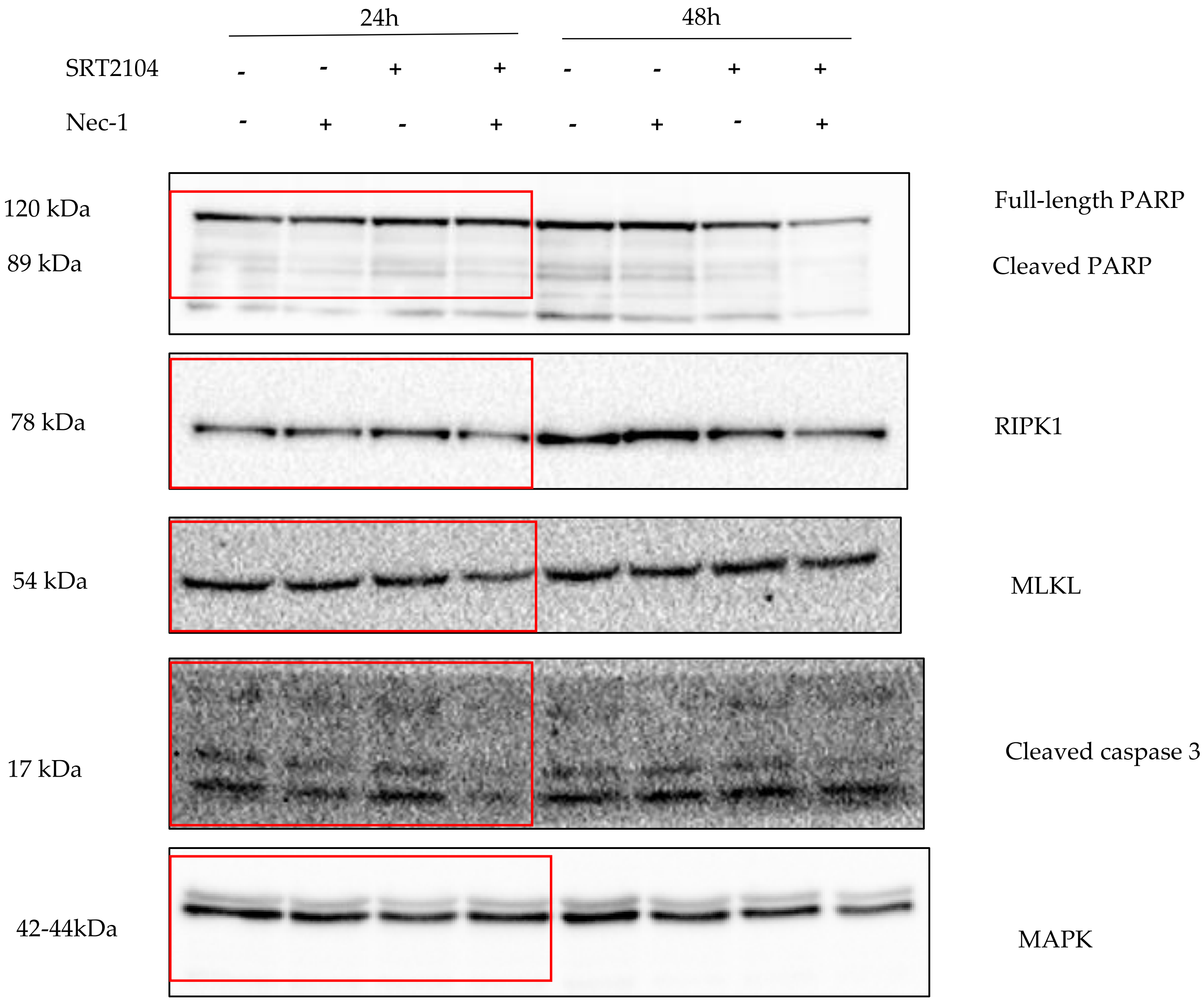

24h
48h
SRT2104 - - + + - - + +
Nec-1 - + - + - + - +
120 kDa
89 kDa
Full-length PARP
Cleaved PARP
78 kDa
RIPK1
54 kDa
MLKL
17 kDa
Cleaved caspase 3
42-44kDa
MAPK
