# Supplementary figures and images for: Diverse actions of sirtuin-1 on ovulatory genes and cell death pathways in human granulosa cells
Source: Reprod Biol Endocrinol. 2022 Jul 15;20:104. doi: 10.1186/s12958-022-00970-x (PMC9284863; doi:10.1186/s12958-022-00970-x)

# SVOG cells

**A**

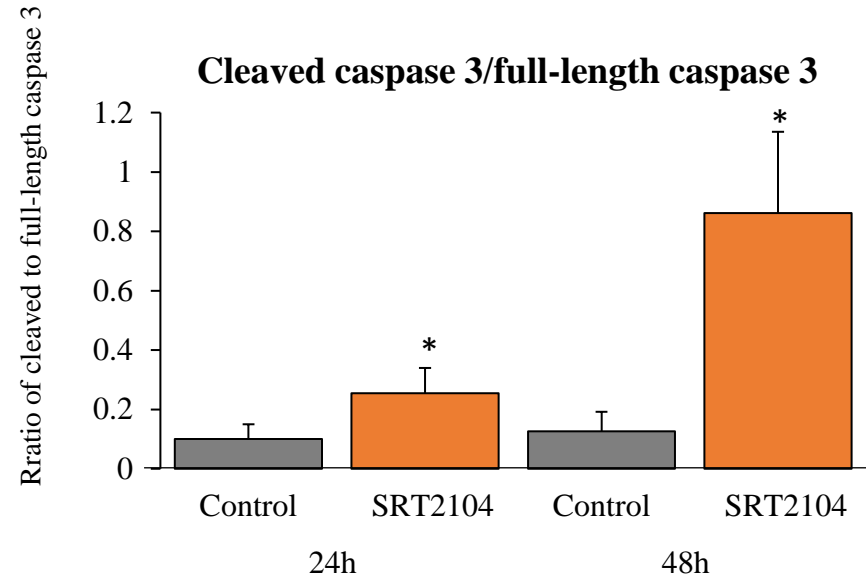

**C**

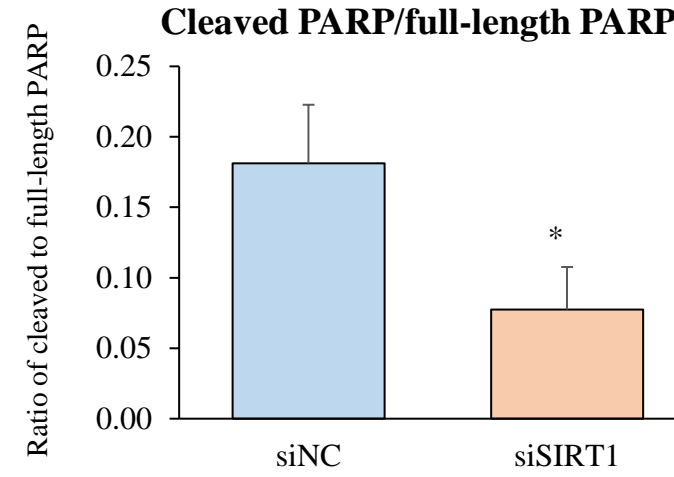

**B**

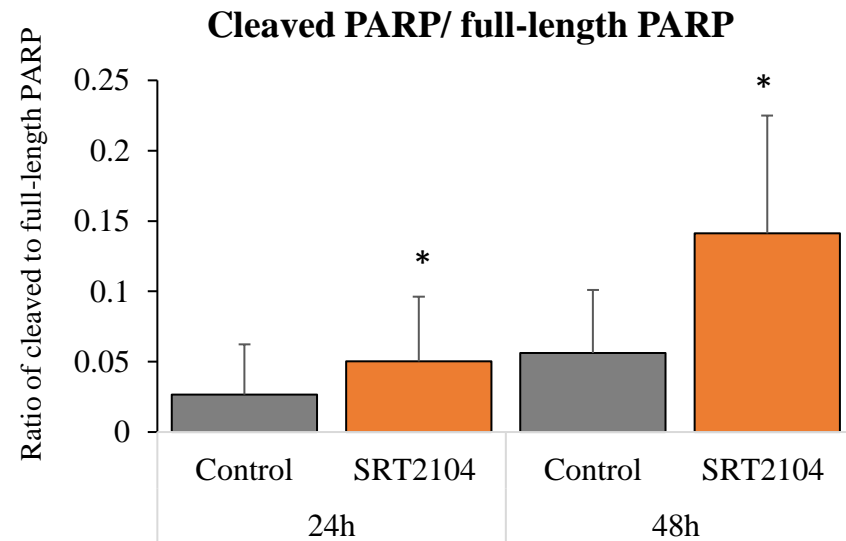

Supplement: Supplementary file 2 — Additional file 2: Supplemental Figure 1. Ratios of cleaved to full length forms of caspase 3 and PARP. (A,B) SVOG cells were treated with either control media or SRT2104 (50 μmol/L) for 24h and 48h. (C) SVOG cells were transfected with 10 nmol/L of either scrambled siRNA (siNC) or SIRT1 siRNA (siSIRT1). The ratios between cleaved to full-length caspase 3 (A) and cleaved to full length PARP (B,C) were calculated from data presented in Figure 6. Asterisks indicate significant differences from their respective controls (*p < 0.05). [file 12958_2022_970_MOESM2_ESM.pdf]
